# Clinicopathologic features of nasopalatine duct cysts: A retrospective study in two Brazilian oral and maxillofacial pathology referral centers

**DOI:** 10.4317/medoral.24718

**Published:** 2021-06-20

**Authors:** Israel Leal Cavalcante, Caio César da Silva Barros, John Lennon Silva Cunha, Vitória Maria Sousa Cruz, Gabriele Alves Pedrosa, Amanda de Jesus Santos, Eveline Turatti, Ricardo Luiz Cavalcanti de Albuquerque-Júnior, Roberta Barroso Cavalcante

**Affiliations:** 1 DDS, MSc. Oral Pathology Section, Department of Dentistry, University of Fortaleza (UNIFOR), Fortaleza, CE, Brazil; 2 DDS, MSc, PhD student. Oral Pathology and Medicine, Postgraduate Program in Dental Sciences, Federal University of Rio Grande do Norte (UFRN), Natal, RN, Brazil; 3 DDS, MSc, PhD student. Oral Pathology Section, Department of Oral Diagnosis, Piracicaba Dental School, University of Campinas (UNICAMP), Piracicaba, SP, Brazil; 4 DDS, MSc student. Oral Pathology Section, Department of Oral Diagnosis, Piracicaba Dental School, University of Campinas (UNICAMP), Piracicaba, SP, Brazil; 5 DDS, University of Fortaleza (UNIFOR), Fortaleza, CE, Brazil; 6 MSc student. Laboratory of Morphology and Experimental Pathology, Institute of Technology and Research, Tiradentes University (UNIT), Aracaju, SE, Brazil; 7 DDS, PhD, Professor. Department of Dentistry, University of Fortaleza (UNIFOR), Fortaleza, CE, Brazil; 8 DDS, PhD, Professor. Laboratory of Morphology and Experimental Pathology, Institute of Technology and Research, Tiradentes University (UNIT), Aracaju, SE, Brazil

## Abstract

**Background:**

Nasopalatine duct cyst (NDC) is the most common non-odontogenic cyst in the oral cavity. Clinically it is not difficult to suspect these lesions based on clinical and radiographic appearance. However, the histopathological diagnosis may be difficult due to the broad morphological diversity of these lesions. The objective was to analyze the clinicopathological features of NDCs diagnosed in two oral and maxillofacial pathology services in the Brazilian northeast.

**Material and Methods:**

A retrospective clinicopathologic study was performed. A total of 18,121 clinical records of oral lesions from two oral and maxillofacial pathology services in Brazil were analyzed (2000-2020). All NDCs cases were revised and demographic, clinical, radiographic, and histopathological data were collected.

**Results:**

Among 18,121 diagnoses in the oral pathology services, 45 (0.2%) were NDCs. The series comprises 24 males (53.3%) and 21 females (46.7%), with a mean age of 43.2 years-old. Most lesions were asymptomatic (n = 27, 60%) with an mean size of 2.1 cm. Microscopically, the non-keratinized stratified squamous epithelium was the most common (66.7%). However, in 88.9% of cases, the epithelial lining was varied and composed of two or more types of epithelium. There was no significant association between the type of epithelium and the size of the cysts (*p* = 0.389). Nerve, blood vessels, hemorrhage, and chronic inflammatory infiltrate were commonly observed. In contrast, there was a low frequency of mucous glands, sebaceous glands, cholesterol clefts, and multinucleated giant cells.

**Conclusions:**

The clinical, radiographic, and microscopic findings observed in this study are similar to those reported in the literature. Due to the morphological diversity of NDC, it is needed to correlate its histopathological features with the clinical and radiographic findings to establish a correct diagnosis.

** Key words:**Oral cavity, oral pathology, nonodontogenic cysts, diagnosis.

## Introduction

Non-odontogenic cysts (NOC) comprise a group of lesions that affect the oral and maxillofacial region. They vary about histogenesis, occurrence, clinicopathological features, biological behavior, and treatment. Among the NOC, the nasopalatine duct cyst (NDC) is the most common and has a reported incidence between 32.8% and 68.8% (1,2). Although the pathogenesis of NDC is still uncertain, it is suggested that its development is due to a probable spontaneous cystic degeneration of the epithelial remnants present in the nasopalatine duct or by the stimulation of these remnants through physical or biological factors, which would cause proliferation and degeneration (3-5).

Generally, NDC is often diagnosed between the fourth and sixth decade of life and exhibits a slight predilection for males (2,3). This lesion is clinically presented as swelling in the anterior region of the maxilla in approximately half of the patients. However, some cases are discovered in the routine clinical and radiographic examination as an incidental finding due to lack of symptoms and slow growth. Also, NDC may exhibit a wide variety of histological features related to both cystic lining epithelium and fibrous capsule (3,5,6). Thus, a proper clinical and radiographic examination and careful histopathological analysis are essential to ensure the correct diagnosis and establish a conservative surgical approach (3,7).

Although several studies have investigated the occurrence and clinicopathological features of NDCs (3-5,8-11), the results have shown slight variations in the prevalence, clinical, and histopathological features in different populations. Thus, the objective of this study was to analyze the clinicopathological features of NDCs diagnosed in two oral and maxillofacial pathology services in the Brazilian northeast to provide a basis for a better understanding of this uncommon lesion.

## Material and Methods

- Study design

In this retrospective cross-sectional clinicopathological study, cases of NDCs diagnosed between January 2000 and June 2020 were retrieved from the archives of two referral Brazilian oral and maxillofacial pathology services: University of Fortaleza (Unifor), Fortaleza, Ceará, Brazil; and Tiradentes University (UNIT), Aracaju, Sergipe, Brazil. Clinical data were collected from the clinical records and evaluated. Cases with data regarding sex, age, location, radiographic characteristics, lesion size, symptomatology, and/or clinical diagnosis were included, while cases without formalin-fixed paraffin-embedded sufficient material to perform the histopathological analysis were excluded.

Histopathological analysis was performed under a light microscope (Olympus CX31, Olympus Japan Co., Tokyo, Japan). Five-micrometer hematoxylin and eosin-stained sections were obtained from each case. Two oral pathologists reevaluated the morphological features of the lesions according to those described by Barros *et al*. (5). Briefly, morphological features of the cystic lining epithelium (epithelium type, presence of goblet cells, and subepithelial hyalinization) and the cystic capsule (inflammatory infiltrate, nerves, blood vessels, presence of hemorrhage, melanin, glandular tissue, cartilage, cholesterol clefts, and multinucleated giant cells) were considered in the microscopic examination.

- Statistical analysis

Data were analyzed and described using the Statistical Package for the Social Sciences 22.0 (SPSS, IBM Corp., Armonk, NY, USA). Continuous variables were expressed as mean and standard deviation. Categorical variables were expressed as the absolute number of cases and percentage values. Fisher's exact test was performed to evaluate the association between the type of cystic lining epithelium and the size of the cyst. The level of significance was set at 5% (*p* ≤ 0.05).

## Results

In the present study, both oral and maxillofacial pathology services received 18,121 surgical specimens between 2000-2020, of which 45 were diagnosed as NDCs (0.2%). As described in Table 1, there was a slightly higher frequency of NDC in male patients (n = 24; 53.3%) (male-to-female ratio of 1.1:1).

**Table 1:**
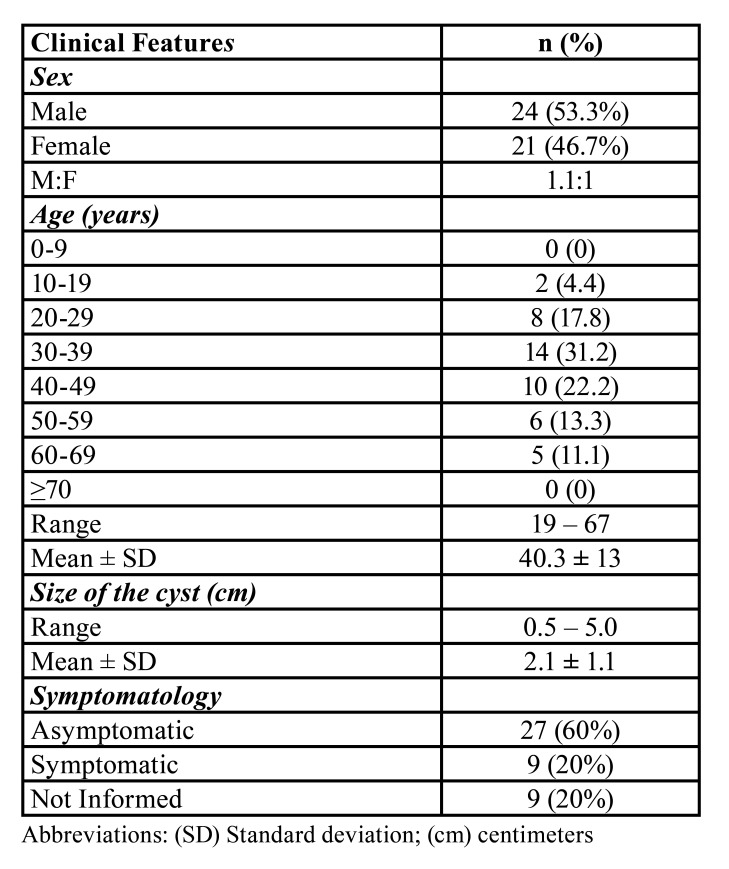
Frequency of clinical features of nasopalatine duct cysts.

NDCs were diagnosed in patients ranging from 19 to 67 years-old and the mean age was 40.3 ± 13 years-old. Patients in the fourth (30-39 years-old) (n = 14; 31.2%) and fifth (40-49 years-old) (n = 10; 22.2%) decade of life were most affected. Clinically, nasopalatine duct cysts presented as swellings of slow growth in the anterior maxilla, sometimes causing the obliteration of the nasolabial fold. Most of cases were asymptomatic (n = 27; 60%), but pain and discomfort had also been mentioned (n = 9; 20%).

Overall, radiographs showed well-defined solitary round or oval radiolucent lesions with sclerotic borders in the periapical region or between maxillary central incisor roots. Occasionally, the lesions exhibited inverted pear-shape or heart-shape due to overlapping of the nasal spine. The lesions ranged from 0.5 to 5.0 cm and had a mean size of 2.1 ± 1.1 cm (Table 1). In most cases, thermal and electrical dental pulp tests were performed to determine pulp vitality and aid in the differential diagnosis. Most lesions were associated with vital teeth (n = 33, 73.3%). Regarding clinical hypothesis, about 46.7% of cases (n = 21) were diagnosed as NDCs. Other presumptive diagnoses had included mainly cystic lesions such as periapical cysts, dentigerous cysts, and nasolabial cysts. Excisional biopsy was performed in 82.2% (n = 37) of the cases analyzed.

**Figure 1:**
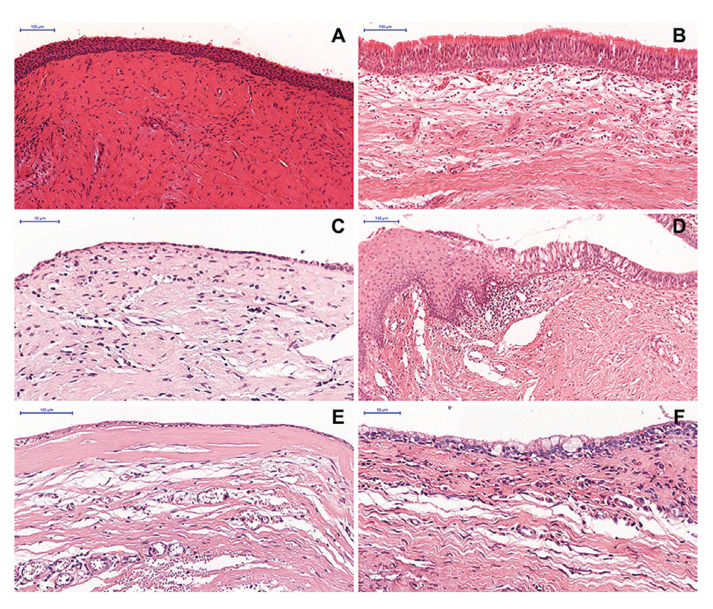
Histopathological features of the epithelial lining of nasopalatine duct cysts (Hematoxylin and Eosin) - Cystic lesion lined by (A - 100 µm) non-keratinized stratified squamous epithelium, (B - 100 µm) ciliated pseudostratified columnar epithelium, and (C - 50 µm) simple cuboidal epithelium. (D - 100 µm) Lesion showing morphological variation in the cystic epithelial lining, highlighting the transition from non-keratinized stratified squamous epithelium to ciliated pseudostratified columnar epithelium. (E - 100 µm) Subepithelial Hyalinization. (F - 50 µm) Goblet Cells.

Morphologically, the epithelial lining of NDCs was classified into two groups according to the type of epithelium predominant. The most frequent type was non-keratinized stratified squamous (n = 30; 66.7%) (Fig. 1) followed by ciliated pseudostratified columnar epithelium (n = 15; 33.3%) (Fig. 1), alone or in combination with other epithelium types (Fig. 1).

Regarding the NDCs cases with a predominance of non-keratinized stratified squamous epithelium, only 1 case (3.3%) was exclusively lined by this epithelium. Most cases were partially lined by a combination of ciliated pseudostratified columnar epithelium and simple cuboidal epithelium (n = 10; 33.3%) (Fig. 1) or only by ciliated pseudostratified columnar epithelium (n = 10; 33.3%). In NDCs with a predominance of ciliated pseudostratified columnar epithelium, only 4 cases (26.6%) were exclusively lined by this epithelium type, 5 cases (33.3%) were partially lined by non-keratinized stratified squamous epithelium, and 5 cases (33.3%) were partially lined by a combination of non-keratinized stratified squamous epithelium and simple cuboidal epithelium (Table 2). Overall, in most cases (n = 40; 88.9%), the epithelial lining varied and was composed of two or more epithelium types (Fig. 1). Subepithelial hyalinization (Fig. 1) and goblet cells (Fig. 1) were observed in 19 (42.2%) and 11 (24.4%) cases, respectively. Statistical analysis did not reveal a significant association between the type of predominant epithelium in the cystic lining and the size of NDCs (*p* = 0.389; OR [95% CI] = 0.47 [0.09-2.33]).

**Figure 2:**
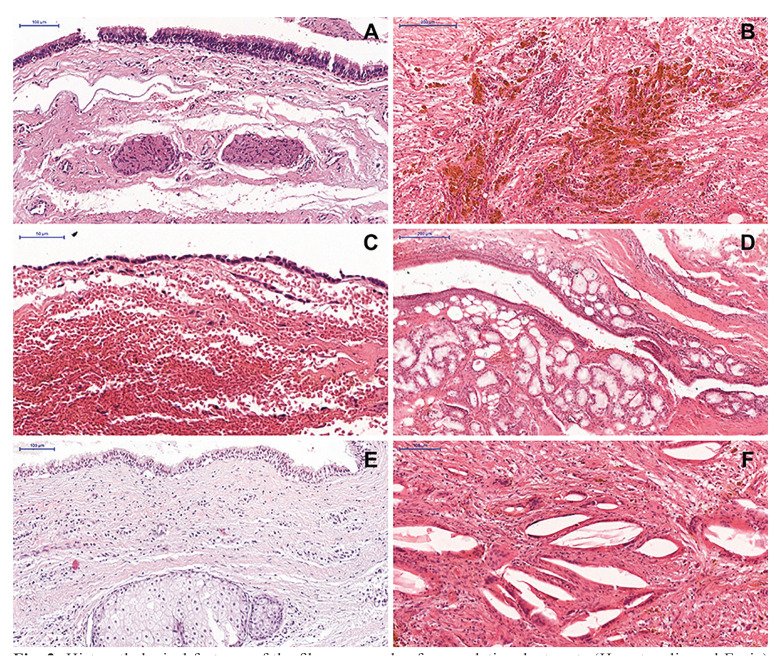
Histopathological features of the fibrous capsule of nasopalatine duct cysts (Hematoxylin and Eosin) - Fibrous cystic capsule showing the presence of (A - 100 µm) nerve, (B - 200 µm) hemosiderin, (C - 50 µm) hemorrhage, (D - 200 µm) mucous glands, (E - 100 µm) sebaceous gland, and (F - 100 µm) cholesterol clefts associated with multinucleated giant cells.

Concerning the cystic capsule components, mild to intense chronic inflammatory infiltrate was observed in most cases (n = 35; 77.8%). Blood vessels (n = 44; 97.8%), hemorrhage (n = 37; 82.2%), nerve fascicles (n = 25; 55.6%), and hemosiderin (n = 16; 35.6%) were also common findings. Besides, salivary mucous glands (n = 8; 17.8%), cholesterol clefts (n = 6; 13.3%), multinucleated giant cells (n = 4; 8.9%), and sebaceous glands (n = 2; 4.4%) were also seen (Fig. 2). Pigmentation by melanin and cartilage was not observed in the fibrous capsule in any of the cases analyzed (Table 2).

**Table 2:**
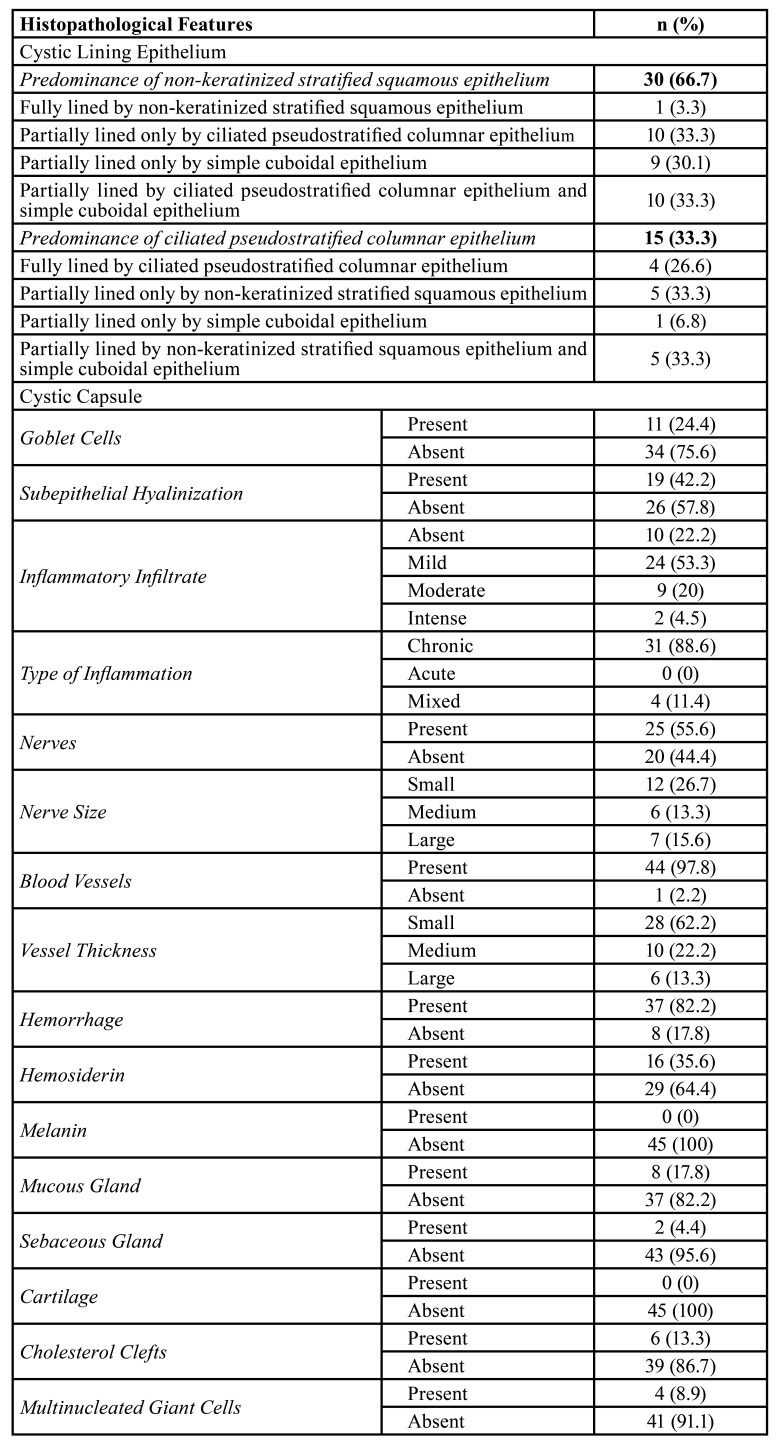
Absolute and relative frequency of histopathological features of nasopalatine duct cysts.

## Discussion

The present study analyzed the occurrence of NDCs in two reference services in the histopathological diagnosis of oral and maxillofacial lesions to contribute to the knowledge about the NDC features. Consequently, the clinical and radiographic characterization performed here may assist the dentist in the clinical diagnosis of this lesion. Besides, by demonstrating the morphological diversity of this lesion, our findings may help the recognition of the structures that comprise the epithelial lining and cystic capsule of the NDC, thus facilitating its histopathological diagnosis by oral pathologists.

The NDC represented 0.2% of the total lesions diagnosed in the referred oral pathology services in the present investigation (2000-2020), similar to previous studies conducted in other oral pathology services (5,12). NDCs can occur at any age, from children to elderly patients; however, they mainly affect adults between the fourth and sixth decade of life (3,8,9,11,13,14), similar to the current study. Although some previous studies do not show sex predilection (3-5,8-10), the current study revealed a slight male predominance (53.3%), with a male-to-female ratio of 1.1:1. Similarly, some studies have reported a higher prevalence of NDC in men (11).

Clinically, NDC may be characterized as an asymptomatic lesion, and this fact may cause its discovery only through routine radiographic examination. Besides, in general, its radiographic images reveal a well-defined rounded or ovoid radiolucent lesion, and it is reported that images with a heart-shape may be less frequent (6,8). These reports are consistent with data obtained in the present study since 60% of the cases were asymptomatic. Also, the lesions were radiographically described as round or oval well-circumscribed radiolucent images with occasional cases exhibiting inverted pear-shape or heart-shape, and mean size of 2.1 ± 1.1 cm. In this way, the obliteration of the nasolabial fold caused by the swelling in the anterior maxilla, as well as the radiographic features observed, may explain the clinical hypotheses observed in the clinical records of our sample since several odontogenic and non-odontogenic lesions may do clinical and radiographic differential diagnosis with nasopalatine duct cyst (8,13).

The most common lesions included in the differential diagnosis of NDCs include inflammatory processes result from pulp necrosis, such as periapical cysts and granulomas. In these circumstances, the pulp vitality test helps rule out lesions of endodontic origin (8). Also, other cysts such as odontogenic keratocysts may present as osteolytic lesions with regular borders in the periapical or interradicular region of anterior teeth. In our study, NDCs were frequently overlooked in the differential diagnosis despite their classic clinical and radiographic features. Only 46.7% of cases were clinically diagnosed as NDCs, demonstrating the unfamiliarity of NDCs among clinicians and dentists. These results emphasize the need to expand differential diagnoses of radiolucent lesions in the anterior maxillary midline and to include NDCs. Interestingly, a differential diagnosis frequently considered by clinicians was the nasolabial cyst (NC). Although these lesions may appear in areas adjacent to the bone at the apex of the root of the incisors, just beneath the lip, they are rare in this location (15). Usually, NCs are often located laterally to the midline in the nasolabial sulcus. Besides, NCs are soft tissue lesions with no bone involvement, and their visualization is not possible in radiographic exams, unlike the NDC (15).

Microscopically, the NDCs are lined by squamous epithelium, ciliated pseudostratified columnar epithelium, cuboidal epithelium, or a combination of these histological types (5,9). In the current study, microscopic analysis of the epithelial lining showed a predominance of non-keratinized stratified squamous epithelium (n = 30, 66.7%). Of these, only one case was exclusively lined by squamous epithelium, and 29 showed a combination with ciliated pseudostratified columnar epithelium and/or simple cuboidal epithelium, similar to previous reports (5). Overall, 88.9% of the cases exhibited a combination of two or more types of epithelium. Although we have not observed a significant association between the type of lining epithelium and the lesion size, it is believed that the type of epithelium is related to the position of the cyst within the incisor canal. Thus, NDCs that develop near the nasal cavity are more likely to exhibit respiratory epithelium features. In contrast, cysts near the oral cavity may exhibit features related to the squamous epithelium (3,5,6,16,17).

Besides that, great morphological diversity of the components of the cystic capsule were observed, such as nerve, vessels, hemorrhage, and hemosiderin, mucous glands, cholesterol clefts, and multinucleated giant cells, similar to previous studies (3,5,8). Although melanin pigmentation and cartilage may be observed in the cystic capsule of CDN (5,8), these findings are quite unusual. Interestingly, sebaceous glands were observed in the fibrous capsule of two cases (0.9%) in the present study. The presence of these glands is uncommon and may also be related to the location of the cyst within the incisor canal and the proximity of the lesion to the oral mucosa.

Some studies have suggested that the NDC emerges from the nasopalatine duct remnants by infections or local trauma (3,14). On the other hand, the absence of inflammatory infiltrate suggests a possible spontaneous cystic formation of NDCs (5). However, most of our cases (n = 35; 77.8%) had a mild to intense inflammatory infiltrate, similar to previous studies (5,6,14,16,17). These findings have led some authors to propose that the NDC develops through mechanisms similar to the periapical cyst. Local stimuli such as inflammation, irritation, or infections could cause the proliferation of epithelial remnants of Malassez and consequently give rise to these lesions (6). However, most NDCs are painless lesions discovered in imaging exams as incidental findings. Also, the patients have not frequently reported trauma history. Another hypothesis suggests that the lesions develop by mechanisms similar to mucus retention cysts and salivary duct cysts related to mucous glands in the maxillary sinus or nasal mucosa (6). The exact etiology of NDCs remains uncertain (5). Further investigations are still necessary to understand and elucidate the pathogenesis of these cysts.

The treatment of choice for NDC is surgical excision and curettage. Also, the size and the positioning of the cyst determine the ideal surgical approach (palatal or labial). However, marsupialization has been proposed as an alternative treatment for large cysts (5,11,18,19). In the current study, most NDCs were treated by excision surgical (excisional biopsy). Perforation to the nasal floor, oro-nasal communication, fistula, injuries to adjacent teeth, infections, hemorrhage, and paresthesia of the anterior palatal region are complications commonly associated with the treatment of this lesion (8,11,16,18). Unfortunately, it was not possible to determine the recurrence rates of the NPDs of the present study due to many cases with incomplete clinical descriptions and lack of follow-up. However, the literature reports recurrence rates relatively low, ranging from 2% to 11% of cases (5).

In summary, the clinical and radiographic findings observed in this study are similar to those reported in the literature. Less than half of the cases were clinically suspected as NDCs, demonstrating little familiarity of dentists with these lesions and the need to include NDCs in the differential diagnoses of radiolucent lesions in the anterior maxillary midline. Non-keratinized stratified squamous epithelium is the most frequent type of epithelium in the NDC cystic lining. In the cystic capsule, nerve, blood vessels, hemorrhage, and chronic inflammatory infiltrate are frequently observed. Due to the varied epithelial lining and the broad morphological characteristics in the fibrous capsule, misdiagnosis may occur. Therefore, pathologists must be aware of the broad morphological diversity of NDC and correlate them with the clinical and radiographic findings to onset a correct diagnosis.
